# Early repolarization pattern associated with coronary artery disease and increased the risk of cardiac death in acute myocardium infarction

**DOI:** 10.1111/anec.12768

**Published:** 2020-05-04

**Authors:** Jun Fan, Feng‐Juan Yao, Yun‐Jiu Cheng, Cheng‐Cheng Ji, Xu‐Miao Chen, Su‐Hua Wu

**Affiliations:** ^1^ Department of Cardiology the First Affiliated Hospital Sun Yat‐Sen University Guangzhou China; ^2^ Key Laboratory of Assisted Circulation NHC Guangzhou China

**Keywords:** acute myocardial infarction, cardiac death, coronary artery disease, early repolarization pattern, eletrocardiography

## Abstract

**Background:**

Early repolarization pattern (ERP) was associated with sudden cardiac death in recent studies. However, the associations between ERP and coronary artery disease (CAD), and ERP and cardiac death caused by acute myocardial infarction (MI) remains unclear.

**Methods:**

We retrospectively enrolled consecutive 1,545 CAD patients and 908 non‐CAD subjects as control group which were confirmed by coronary angiograph. The CAD patients include stable CAD, acute MI patients, and old MI patients. Multivariate logistic regression was employed to evaluate the relationship between ERP and CAD, and ERP and cardiac death caused by acute MI.

**Results:**

Of the 1,545 CAD subjects, there were 1,029 stable CAD patients, 404 acute MI patients, and 112 old MI patients. The incidence of ERP was much higher among patients with CAD than without CAD subjects (20.1% vs. 6.2%, *p* < .001) after adjusting for major cardiovascular risk factors. No significant correlation was observed between lead region of ERP on 12‐lead ECG and single abnormal artery. Of the 404 acute MI patients, 342 patients survived and 62 patients died. Incidence of ERP was higher in non‐survivor than survivor patients with acute MI (24.2% vs. 17.5%, *p* = .006) after adjustment for major cardiovascular risk factors.

**Conclusion:**

The incidence of ERP was higher in CAD patients than subjects without CAD and in non‐survivor patients than survivor patients with acute MI. The lead region of ERP on 12‐lead ECG was not associated with single abnormal coronary artery.

## 1 INTRODUCTION

Early repolarization pattern (ERP), which is characterized by the slurring or notching J wave at the end QRS complex in at least two consecutive leads on the electrocardiogram, is common among young, healthy individuals (Aagaard et al., 2014). Recent studies showed that ERP is a genetic mutation‐related ECG morphology (Barajas‐Martinez et al., 2012; Guo et al., 2016; Liu et al., 2017). However, clinical observational studies showed that incidence of ERP in CAD patients was 9%–28.6% (Lee et al., 2014; Naruse et al., 2014; Park et al., 2014), which was much higher than incidence of ERP among general population (Aagaard et al., 2014). This phenomenon partly contradicted the opinion that ERP is caused by gene mutation. The relationship between ERP and CAD remains unclear.

Early repolarization pattern has attracted attention for its association with sudden cardiac death (SCD) among population without structural heart disease (Pieroni, Bellocci, & Crea, 2008). Coronary artery disease (CAD) is one of important risk factors for SCD, and some recent studies concluded that ERP is also a risk factor for SCD among CAD patients (Lee et al., 2014; Naruse et al., 2014; Park et al., 2014; Seo et al., 2017). At the same time, most studies regarding CAD and ERP reported only the association between the ERP and risk for death from arrhythmia or cardiac cause with routine follow‐up (Lee et al., 2014; Naruse et al., 2014; Park et al., 2014; Seo et al., 2017), and little is known about whether ERP is a risk factor for hospital death caused by acute myocardial infarction (MI).

Therefore, we conducted a study to investigate whether CAD patients were prone to show ERP on 12‐lead ECG than subjects without CAD, and the association between ERP and cardiac death caused by acute MI among hospitalized CAD patients.

## 2 METHODS

### 2.1 Study design

The study was a retrospective case–control study. The study was conducted with the consent of the ethics committee of the first affiliated hospital of Sun Yat‐Sen University. The subjects of this study were all patients who were hospitalized in first affiliated hospital of Sun Yat‐Sen university from 2011 to 2013. All participants provided written informed consent to participate in the study.

We included CAD patients contain stable CAD patients, acute MI patients, and old MI patients. To clarify the issue more clearly, unstable angina patients were excluded. CAD was defined as coronary artery stenosis which is >50% confirmed by coronary angiography (CAG). The results of CAG were analyzed by two cardiologists through discussion. For those patients who refused and not suitable for CAG, acute MI was diagnosed based on the patient's symptoms, ECG, and cardiac troponin. The definition of acute MI has been described previously (Thygesen et al., 2012). The old MI subjects were patients who experienced MI over 4 weeks. By design, in addition to CAD, other structural heart disease and pericardial diseases were excluded (confirmed by echocardiography, CAG, and ventricular angiography). Subjects who had a specific electrocardiogram (as mentioned below) were also excluded. Subjects without CAD were patients whose coronary artery stenosis which is <50% confirmed by CAG and who had no other structural heart disease. Patients with and without CAD were selected to investigate the relationship between CAD and ERP. As a result, a total of 2,453 subjects were included.

Patients who had single coronary artery stenosis were selected to investigate the relationship between the ECG lead region of ERP and abnormal coronary artery. Patients were divided into three groups: left anterior descending coronary artery (LAD) stenosis, right coronary artery (RCA) stenosis, and left circumflex artery (LCX) stenosis. As a result, a total of 138 patients were included.

Patients who had acute MI and discharged alive and patients died of acute MI were selected to study the association between cardiac death and ERP. As a result, a total of 62 patients died of acute MI and 342 surviving patients were included.

### 2.2 ECG characteristic analysis

All ECG of patients during their hospitalization were assessed. The ECG of the patients was collected once they admitted in hospital. The ECGs were assessed by two cardiologists who did not know the patients’ clinical characteristics and group. Patients with ventricular tachycardia (VT), complete left bundle branch block, complete right bundle branch block, left anterior fascicular block, left posterior fascicular block, WPW syndrome, nonspecific intraventricular conduction delay, and Brugada pattern were excluded. ERP was defined as that J point raised above baseline by more than 0.1 mV with slurring or notching changes in the terminal of the QRS complex wave in at least two ECG leads. As defined by prior studies (Oh et al., 2013), notching was noted as a positive deflection at the terminal portion of a positive QRS complex. Slurring was defined as a smooth transition from QRS complex to ST segment with upright concavity. The morphology and lead position of the ERP were investigated. If the results of the ECG were in dispute, the final results were judged by an expert in ECG.

### 2.3 Statistical analysis

Baseline characteristics of participants are presented as means and standard deviations for continuous variables and percentages for categorical variables. Comparison of continuous variables between cases and control subjects was performed with Student's *t* test for paired data. Categorical variables were compared using a chi‐square test of Fisher's exact test, as appropriate among groups. The effects of covariates on CAD and cardiac death were analyzed using multivariate logistic regression. Statistical analyses were conducted using SPSS version 22 (IBM Corp.). In 2‐sided tests, a *p*‐value < .05 was considered statistically significant.

## 3 RESULTS

### 3.1 General clinical features

Among the 3,271 patients enrolled in the study, 2,453 fulfilled the inclusion criteria. The patients were divided into CAD group and non‐CAD group. The basic clinical characteristics of patients with and without CAD were showed in Table 1. We next selected out the included acute MI patients who died of acute MI in hospital and who discharged alive as control group to observe the relationship between ERP and death in patients with acute MI. Finally, 404 patients fulfilled the inclusion criteria for the present study and were included in the study. We compared characteristics of their basic clinical data and ECG in Table 2.

**TABLE 1:** Clinical characteristics of the included patients

	With CAD	Without CAD	*p*	Adjust *p* ^a^	OR	95% CI
	*n* = 1,545	*n* = 908				
Age, year	61.86 ± 11.60	60.27 ± 10.41	<.001	<.001	1.034	1.02–1.04
Male, no. (%)	1,161 (75.1)	476 (52.4)	<.001	<.001	1.838	1.40–2.42
Smoking, no. (%)	610 (39.5)	210 (23.1)	<.001	.002	1.550	1.17–2.05
LVEF, %	63.97 ± 11.49	68.56 ± 7.84	<.001	<.001	0.961	0.95–0.97
Diabetes, no. (%)	383 (24.9)	148 (16.3)	<.001	.001	1.623	1.21–2.17
Hypertension, no. (%)	882 (57)	535 (58.9)	.012	.05	0.787	0.62–1.00
TC, mM	4.47 ± 1.19	4.58 ± 1.12	.140	.001	0.630	0.48–0.82
LDL‐C, mM	2.98 ± 1.15	2.96 ± 1.00	.308	<.001	1.813	1.38–2.39
HDL‐C, mM	1.05 ± 0.48	1.16 ± 0.13	<.001	.01	0.673	0.50–0.91
ERP, no. (%)	311 (20.1)	56 (6.2)	<.001	<.001	3.834	2.62–5.60

Abbreviations: CAD, coronary artery disease; ERP, early repolarization pattern; HDL‐C, high‐density lipoprotein cholesterol; LDL‐C, low‐density lipoprotein cholesterol; LVEF, left ventricular ejection fraction; TC, total cholesterol.

^a^ Multivariate logistic regression analysis adjusted for age, sex, smoking status, diabetes, hypertension, and LVEF.

**TABLE 2:** Clinical Characteristics of Survivors and Non‐survivors with AMI

	Survivors	Non‐survivors	*p*	Adjusted *p* ^a^	OR	95% CI
	*n* = 342	*n* = 62				
Age, year	61.77 ± 11.15	73.65 ± 11.47	<.001	<.001	1.736	1.47–4.61
Male, no. (%)	277 (81.0)	29 (46.8)	<.001	<.001	0.132	0.04–0.41
Smoking, no. (%)	172 (50.3)	14 (22.6)	.020	.498	1.440	0.50–4.13
Diabetes, no. (%)	87 (25.4)	19 (30.6)	.169	.313	1.584	0.65–3.87
Hypertension, no. (%)	169 (49.4)	35 (56.5)	.150	.260	0.595	0.24–1.47
Killip class, no. (%)						
Killip I	223 (65.2)	11 (17.7)	<.001	<.001		
Killip II	76 (22.2)	19 (30.6)				
Killip III	23 (6.7)	11 (17.7)				
Killip IV	20 (5.8)	21 (33.9)				
ERP (+), no. (%)	60 (17.5)	15 (24.2)	.095	.006	4.219	1.52–11.68
ERP Leads, no. (%)						
Inferior leads (II, III, aVF)	39 (11.4)	12 (19.4)	.044	.009	3.711	1.92–8.46
Lateral limb leads (I, aVL)	6 (1.8)	3 (4.8)	.472	.871	1.357	0.53–13.64
Precordial leads (V1–V6)	15 (4.3)	4 (6.5)	.295	.840	0.671	0.35–19.18
ERP morphology, no. (%)						
Slurring	48 (14.0)	17 (27.4)	.762	.247	5.410	0.81–27.45
Notching	23 (6.7)	6 (9.7)	.469	.889	1.193	0.60–15.28

Abbreviations: AMI, acute myocardium infarction; ERP, early repolarization.

^a^ Multivariate logistic regression analysis adjusted for age, sex, smoking status, diabetes, hypertension, and Killip class.

### 3.2 Relationship between ERP and CAD

**FIGURE 1:**
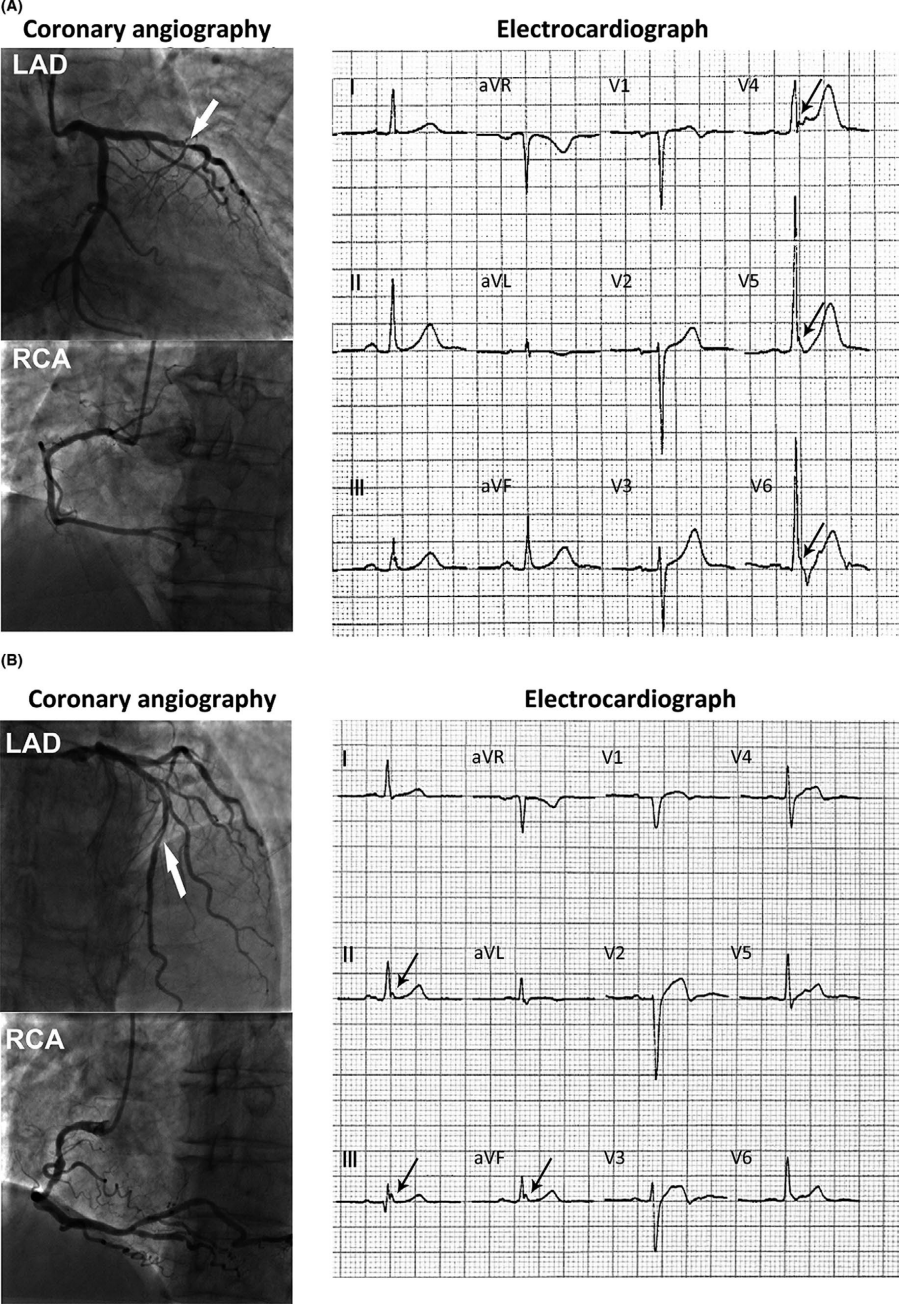
Two CAD patients with ERP and LAD lesion. (a) A CAD patient with LAD lesion (>50%, white arrow). The 12‐lead ECG showed ERP in V4–V6 (black arrows). (b) A CAD patient with LAD lesion (>50%, white arrow). The 12‐lead ECG showed ERP in Ⅱ, Ⅲ, and aVF (black arrows). CAD, coronary artery disease; ERP, early repolarization pattern; LAD, left anterior descending coronary artery

**FIGURE 2:**
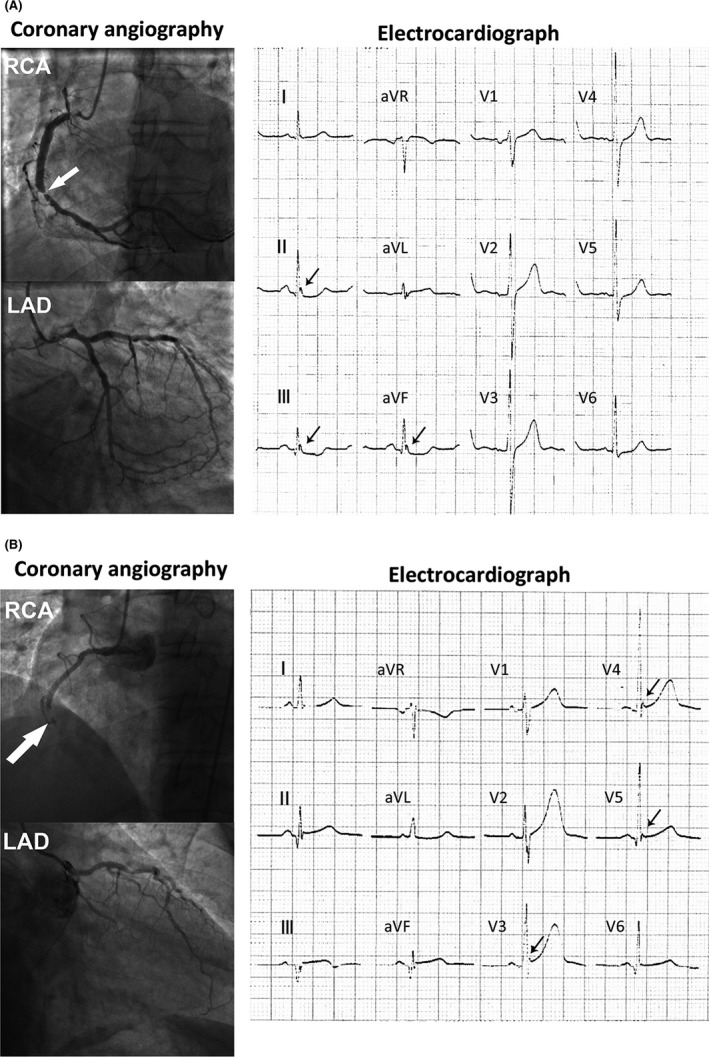
Two CAD patients with ERP and RCA lesion. (a) A CAD patient with RCA lesion (>50%, white arrow). The 12‐lead ECG showed ERP in Ⅱ, Ⅲ, and aVF (black arrows). (b) A CAD patient with RCA completely occlusion (white arrow). The 12‐lead ECG showed ERP in V3–V5 (black arrows). CAD, coronary artery disease; ERP, early repolarization pattern; RCA, right coronary artery

The incidence of ERP was higher in CAD patients than non‐CAD patients (20.1% vs. 6.2%) (Table 1). CAD patients have significant differences in age, gender, smoking, diabetes, LVEF, LDL‐c, and HDL‐c compared with non‐CAD patients. ERP was associated with CAD after adjusted cardiovascular factors (*p* < .001, aOR = 3.834, 95%CI = 2.62–5.60) compared with no‐CAD patients. Four patients with coronary artery lesion (>50%) and ERP were shown in Figures 1 and 2. We also compared the occurrence of ERP in different types of CAD patients (Table 3). Compared with patients without CAD, ERP was associated with stable coronary disease and acute MI after adjustment for major cardiovascular risk factors (both *p* < .001). The frequency of ERP in old MI was higher than non‐CAD cases. However, ERP was not associated with old MI (*p* = .089). When morphology of ERP, notching, or slurring were analyzed, slurring of the terminal portion of QRS was more common in both CAD patients and non‐CAD patients (Table 4). There was no significant difference in morphology among patients with and without CAD after adjustment for cardiovascular risk factors. With respect to ECG lead region of ERP, inferior leads were more common in both CAD patients and non‐CAD patients. There was no difference in the ECG lead region of the ERP between CAD patients and non‐CAD patients before and after statistical adjustment for coronary risk factors.

**TABLE 3:** Distribution of ERP in patients with CAD

	ERP (+)	ERP (−)	*p* ^a^	Adjusted *p* ^a^, ^b^
Non‐CAD patients, no. (%)	56 (6.2)	852 (93.8)		
CAD patients, no. (%)	311 (20.1)	1,234 (79.9)		
Stable coronary artery disease, no. (%)	230 (22.3)	799 (77.6)	<.001	<.001
Acute MI, no. (%)	68 (16.8)	336 (83.2)	<.001	.002
Old MI, no. (%)	13 (11.6)	99 (88.4)	.108	.089

Abbreviations: CAD, coronary artery disease; ERP, early repolarization pattern; MI, myocardial infarction.

^a^ Compared with no‐CAD patients.

^b^ Multivariate logistic regression analysis adjusted for age, sex, smoking status, diabetes, hypertension, and LVEF.

**TABLE 4:** ECG Characteristics of Patients with and without CAD

	With CAD	Without CAD	*p*	Adjusted *p* ^a^	OR	95% CI
	*n* = 1,545	*n* = 908				
ERP (+), no. (%)	311 (20.1)	56 (6.2)				
ERP leads, no. (%)						
Inferior leads (II, III, aVF)	227 (14.7)	49 (5.4)	.325	.437	1.455	0.57–3.75
Lateral limb leads (I, aVL)	30 (1.9)	1 (0.1)	.187	.494	0.472	0.06–4.06
Precordial leads (V1–V6)	68 (4.4)	9 (1.0)	.697	.507	0.720	0.27–1.91
ERP morphology, no. (%)						
Slurring	244 (15.8)	44 (4.8)	.220	.642	0.823	0.32–1.87
Notching	135 (8.7)	27 (3.0)	.598	.622	1.205	0.58–2.53

Abbreviations: CAD, coronary artery disease; ERP, early repolarization pattern.

^a^ Multivariate logistic regression analysis adjusted for age, sex, smoking status, diabetes, hypertension, and LVEF.

### 3.3 Association between ECG regional leads of ERP and abnormal coronary artery

In order to illustrate the relationship between abnormal coronary arterial territory and localization of ERP in ECG, we enrolled 138 ERP (+) subjects with single coronary artery stenosis confirmed by CAG. When region of ERP leads was analyzed in patient with single coronary artery stenosis, there was no significant difference between localization of ERP in ECG and abnormal artery (Table 5). Two patients with single LAD stenosis, respectively, presented inferior leads ERP (Figure 1a) and precordial leads ERP (Figure 1b). Another two patients with RCA stenosis, respectively, presented inferior leads ERP (Figure 2a) and precordial leads ERP (Figure 2b). ERP was noted more frequently in inferior leads among three groups.

**TABLE 5:** ECG lead region of ERP and location of single coronary artery stenosis

	Single coronary artery stenosis patients with ERP			*p*
	RCA (*n* = 107)	LAD (*n* = 437)	LCX (*n* = 67)	
ERP patients, no. (%)	37 (34.5)	87 (19.9)	14 (20.9)	<.001
ECG lead region of ERP				
Inferior leads (II, III, aVF), no. (%)	28 (26.1)	70 (16.0)	11 (16.4)	.695
Lateral limb leads (I, aVL), no. (%)	4 (3.7)	5 (1.1)	1 (1.4)	
Precordial leads (V_1_–V_6_), no. (%)	5 (4.7)	21 (4.8)	3 (4.5)	

Note

One hundred and thirty‐eight CAD patients with single coronary artery stenosis and ERP were included to study the relationship between location of abnormal artery and region of ERP leads on ECG.

Abbreviations: CAD, coronary artery disease; ERP, early repolarization pattern; LAD, left anterior descending coronary artery; LCX, left circumflex artery; RCA, right coronary artery.

### 3.4 Relationship between ERP and death of acute MI

The incidence of ERP was higher in non‐survivor patients than survivor patients with acute MI (24.2% vs. 17.5%) (Table 2). ERP was not associated with cardiac death caused by acute MI before adjustment for main cardiac risk factors but was after adjustment for main cardiac risk factors (Table 2). When ECG regional leads and morphology of ERP were analyzed, slurring‐like ERP and inferior leads of ERP were noted more common in both survivors and non‐survivors with acute MI. Inferior region of ERP was associated with death caused by acute MI compared with survivors after adjusted by major cardiovascular risk factors (*p* = .009, aOR = 3.711, 95%CI = 1.92–8.46). However, the type of ERP (slurring or notching) and the other regional leads of ERP (inferior and lateral) did not differ significantly between survivors and non‐survivors with acute MI.

## 4 DISCUSSION

In present study, we found that the incidence of ERP on 12‐lead ECG was higher in CAD patients than non‐CAD patients, before and after adjusting for major cardiovascular risk factors. There was no significant difference in ECG morphology and lead region of ERP between patients with and without CAD. The incidence of ERP was higher in non‐survivor patients than survivor patients with acute MI after adjusting for cardiovascular risk factors.

Previous studies showed that ERP is common among young, healthy individuals. Several genetic mutations, including CACNA1C (Liu et al., 2017), SCN5A (Guo et al., 2016), and KCNJ8 (Barajas‐Martinez et al., 2012), had been identified to be related to ERP. ERP was considered to be a gene‐related morphology which was similar to Brugada syndrome (Antzelevitch & Yan, 2011). Recent studies showed the incidence of ERP in patients with CAD was 9%–28.6% (Lee et al., 2014; Naruse et al., 2012; Park et al., 2014), significantly higher than the incidence of ERP in general population (Aagaard et al., 2014). Suh et al. (2016) found that ERP was associated with asymptomatic CAD and regional leads of ERP was not associated with the location of coronary artery stenosis, which was verified by coronary computed tomography angiography (CCTA).

In our study, CAD was diagnosed by a more accurate method, CAG, rather than CCTA. We found that incidence of ERP among CAD patients was higher than non‐CAD patients (20.1% vs. 6.2%, *p* < .001). At the same time, we found that the lead region of ERP was not associated with arterial territory of significant stenosis. The frequency of ERP in old MI is higher than non‐CAD subjects, but there was no significant difference between the two groups in our study. This result may be interpreted by the reason that the number of old MI is relatively small.

The association between ERP and adverse cardiovascular outcomes in patients with acute MI remains controversial. Some studies reported that ERP was associated with increased risk of cardiac death in patients with acute MI. In a propensity score‐adjusted cohort study, the presence of ERP had a multivariable‐adjusted association with increased risk of sustained VT or ventricular fibrillation (VF) in patients with anterior ST‐segment elevation myocardial infarction (STEMI) in the early 30 days (Chen et al., 2017). Another case–control study indicated that ERP was associated with increased risk of ventricular arrhythmia in the setting of acute STEMI (Ali Diab, Abdel‐Hafez Allam, Mohamed, Mohamed, & Abel‐Hafeez Khalid, 2015). In Naruse's retrospective study, the presence of ERP increased the risk of VF occurrences within 48 hr after acute MI onset (Naruse et al., 2012). However, a cohort study suggested that ERP was not associated with an increased risk of SCD, CAD death, or cardiovascular mortality (O'Neal et al., 2016).

We found that the incidence of ERP was higher in non‐survivor patients than survivor patients with acute MI after adjustment for confounding factors. As acute MI is an important cause of death in patients with CAD, it is crucial to assess the risk factors for hospital death in patients with acute MI. Our study suggested that ERP may be one of risk factors for cardiac death among patient with acute MI during hospitalization. We propose a possible mechanism: The mechanism of ERP mainly involves the dynamical difference of transient outward potassium current (I_to_) between endocardium and epicardium (Antzelevitch, 2012). The ventricular epicardium commonly displays action potential (AP) with a prominent transient outward K^+^ current‐mediated notch (spike and dome). The absence of a prominent AP notch in the endocardium is the consequence of a much smaller I_to_. An I_to_‐mediated AP notch in the epicardium, but, not endocardium, may produce a transmural voltage gradient during early ventricular repolarization that could manifest as ERP on the ECG (Yan, Lankipalli, Burke, Musco, & Kowey, 2003). Therefore, factors that influence I_to_ or its counter (inward) currents in phases 1 and 2 (e.g., hypothermia [Federman, Mechulan, Klein, & Krahn, 2013], heart rate [Stern, 2014] and ion channel disease [Antzelevitch & Yan, 2011]) could modify the size and morphology of the ERP. Recent study showed that I_to_ was increased by ischemia (Yan et al., 2004). The increased I_to_ due to ischemia increased the transwall voltage gradient in early ventricular repolarization significantly. So, patients with CAD were prone to show ERP. However, our results showed that there was no significantly difference between abnormal coronary artery and ECG lead region of ERP, which was similar as recent study (Suh et al., 2016). This may suggest that other mechanisms may be involved in the ERP occurrence in patients with CAD.

The phase‐2 re‐entry caused by heterogeneity of repolarization between endocardium and epicardium is the main mechanism that ERP (+) subjects tend to develop VT/VF (Antzelevitch, 2012). As well known, acute MI is a very important risk factor for initiation of VT/VF. Therefore, it is not difficult to understand that acute MI patients with ERP may be more prone to develop VT/VF after adjustment for other cardiac risk factors.

This study has some limitations. First, the elevation of J point may be confused by ST‐segment elevation in patients with CAD, which may be a reason for the increased ERP ratio in patients with CAD. Second, most of the non‐CAD patients enrolled in this study had chest pain. Although the incidence of ERP in this population was similar with general population, other characteristics might be different, which may result in differences in statistical results. Third, when ERP was verified as a risk factor for hospitalized cardiac death caused by acute MI, the number of deaths was relatively small. Fourth, this study was a retrospective case–control study. It was difficult to guarantee the authenticity of information, and it was difficult to judge whether CAD was the etiology of ERP.

In conclusion, the incidence of ERP in patients with CAD was higher than that of patients without CAD. The lead region of ERP on ECG was not associated with single abnormal coronary artery. The incidence of ERP was more common in non‐survivor patients than survivor patients with acute MI.

## CONFLICTS OF INTEREST

The authors have no other funding, financial relationship, or conflicts of interest to disclose.

## AUTHOR CONTRIBUTIONS

WSH and FJ made substantial contributions to the design of the present study. FJ, JCC, CYJ, and CXM performed the experiments; FJ, JCC, CYJ, and CXM analyzed the data; FJ and WSH wrote the manuscript. All authors have read and approved the final version of the manuscript.

## ETHICAL APPROVAL

The study was a retrospective case–control study. The study was conducted with the consent of the ethics committee of the first affiliated hospital of Sun Yat‐Sen University. The subjects of this study were all patients who were hospitalized in first affiliated hospital of Sun Yat‐Sen university from 2011 to 2013.
